# Supplementary material for: Task-specific modulation of corticospinal neuron activity during motor learning in mice
Source: Nat Commun. 2023 May 11;14:2708. doi: 10.1038/s41467-023-38418-4 (PMC10175564; doi:10.1038/s41467-023-38418-4)
Supplement: Supplementary file 1 — Supplementary information [file 41467_2023_38418_MOESM1_ESM.pdf]

Supplementary Information for: Task-specific modulation of corticospinal neuron activity during motor learning in mice

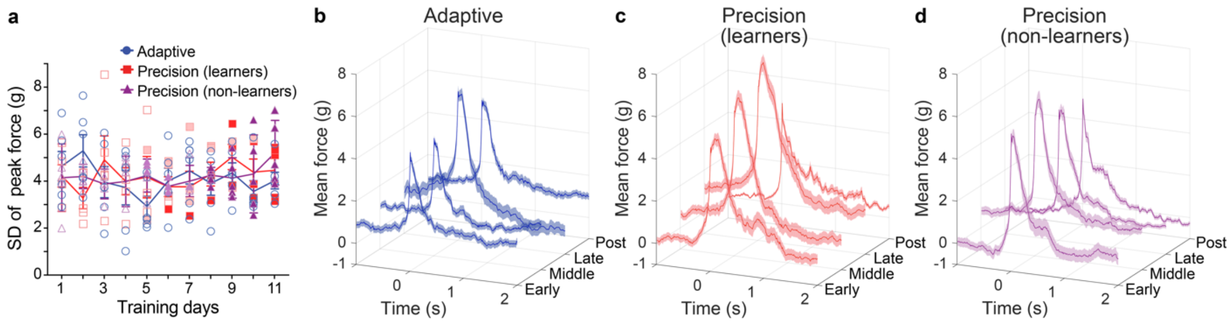

Supplementary Figure 1. Pull force and kinetics in head-fixed mice. a. Standard deviation of mean pull force during the first 11 days of head-fixed behavioral training. On precision pull, open squares and triangles are data from phase 1 (5-20g), light shaded from phase 2 (8-20g), and saturated from phase 3 (13-19g). No significant differences were detected between tasks. **b-d.** Mean pull kinetics (mean pull force over time) in head-fixed mice during the 2 second reward window (starts at time 0). Mice that learned precision pull show refined movement with training that is disrupted by pyramidotomy. All data are presented as mean ± s.e.m., *n* = 5 mice/group.

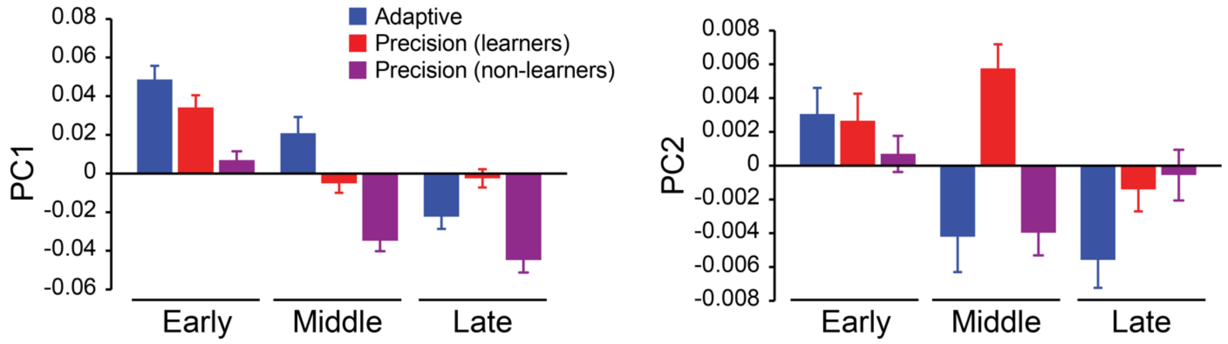

Supplementary Figure 2. First two principal components from PCA of corticospinal neuron activity during task acquisition. Data presented as mean ± s.e.m., *n* = 11,808 individual cells and trials (early, skilled), 14,208 (middle, skilled), 16,944 (late, skilled), 9840 (early, adaptive), 6,480 (middle, adaptive), 9,360 (late, adaptive), 21,840 (early, non-learners), 12,672 (middle, non-learners), and 9,120 (late, non-learners).

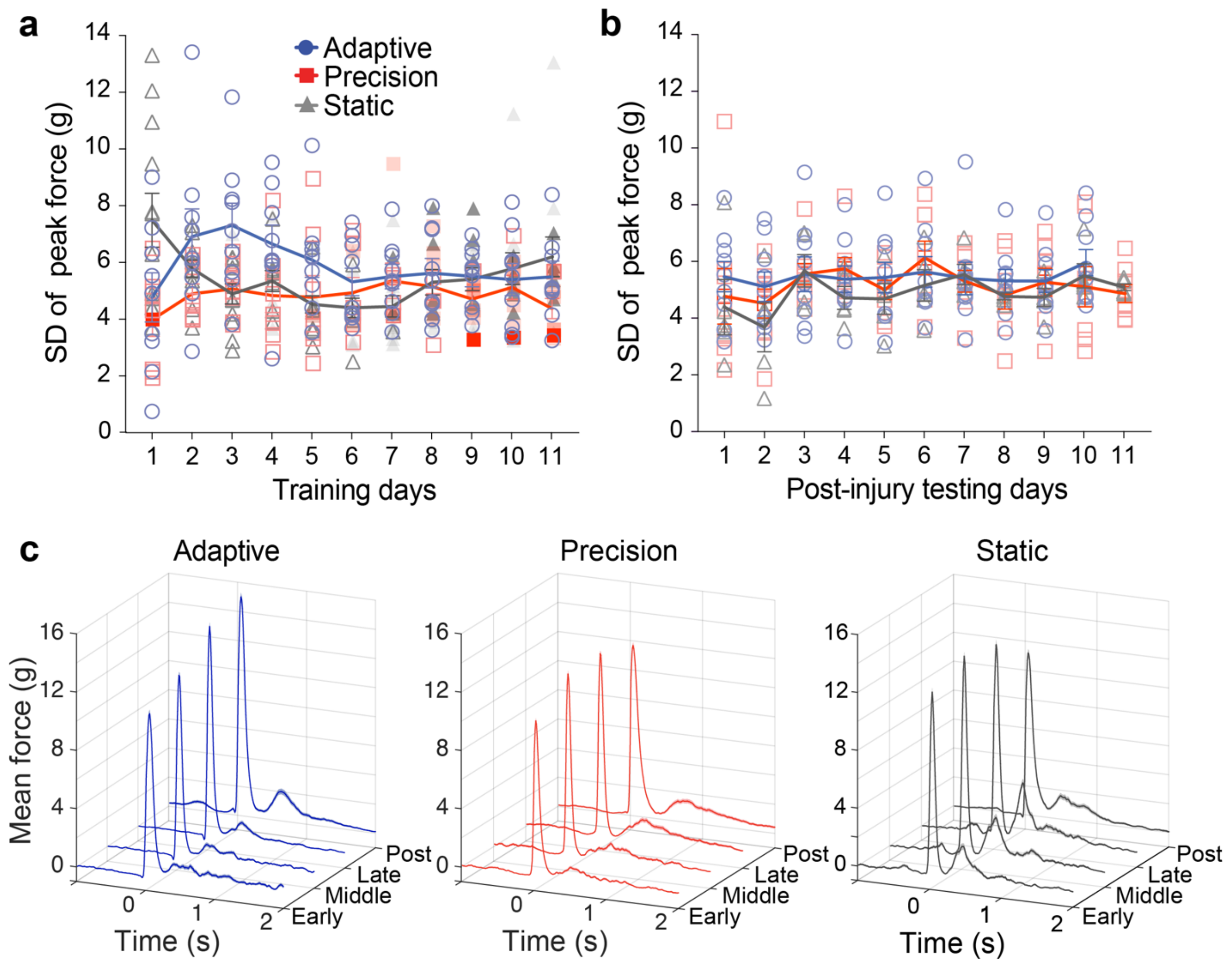

Supplementary Figure 3. Pull force in freely moving behavior. a. Standard deviation of mean pull force during the first 11 days of freely moving behavioral training. On precision pull, open squares and triangles are data from phase 1 (5-20g), light shaded from phase 2 (8-20g), and saturated from phase 3 (13-19g). No significant differences were detected between phases. **b.** Standard deviation of mean pull force during the first 11 days of testing post-injury. **c.** Mean pull kinetics (mean pull force over time) in freely moving mice during the 2 second reward window (starts at time 0). All data are presented as mean ± s.e.m., in panels **a,c** for *n* = 9 mice (adaptive), 8 (precision), and 12 (static, except post-injury panel c); and in panel **b** for *n* = 9 (adaptive), 8 (precision), and 5 (static, and in post-injury panel c).

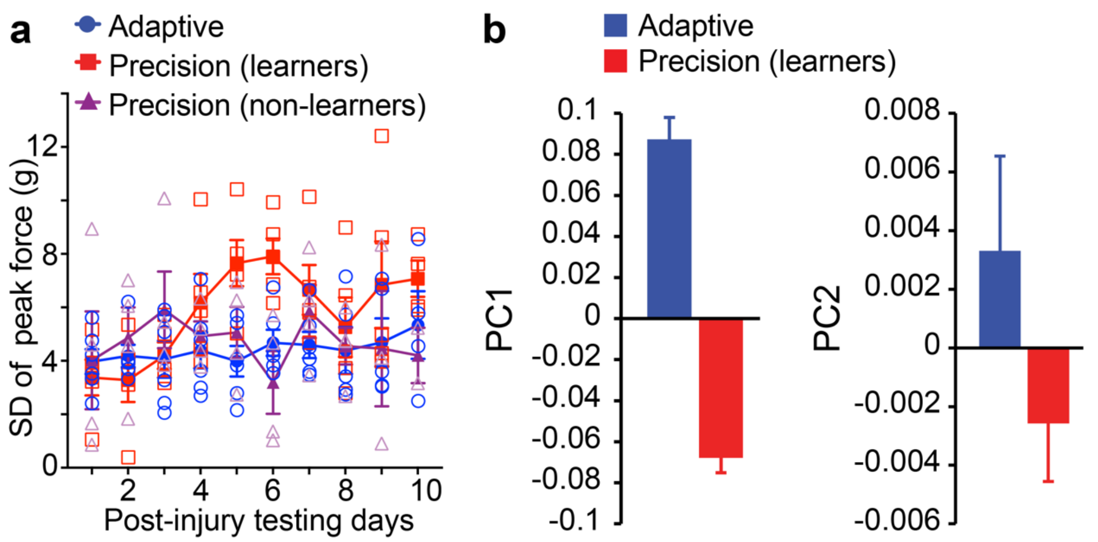

Supplementary Figure 4: Precision isometric pull in head-fixed mice is disrupted by pyramidotomy. a. Standard deviation of peak pull force during the 10 post injury days of head-fixed behavioral testing. Precision and adaptive groups showed the same rate of testing-related change (common regression slope = 0.3 g/day, although the injury significantly impaired the precision group as shown by the significant difference in the intercept ($F_{1,92}$ = 8.0, P = 0.006, n = 5/group). **b.** First two principal components from PCA of corticospinal neuron activity after pyramidotomy. Data presented as mean ± s.e.m., in panel **a** n = 5 mice/group and in panel **b** n = 5,400 individual cells and trials (skilled) and 4,200 (adaptive).

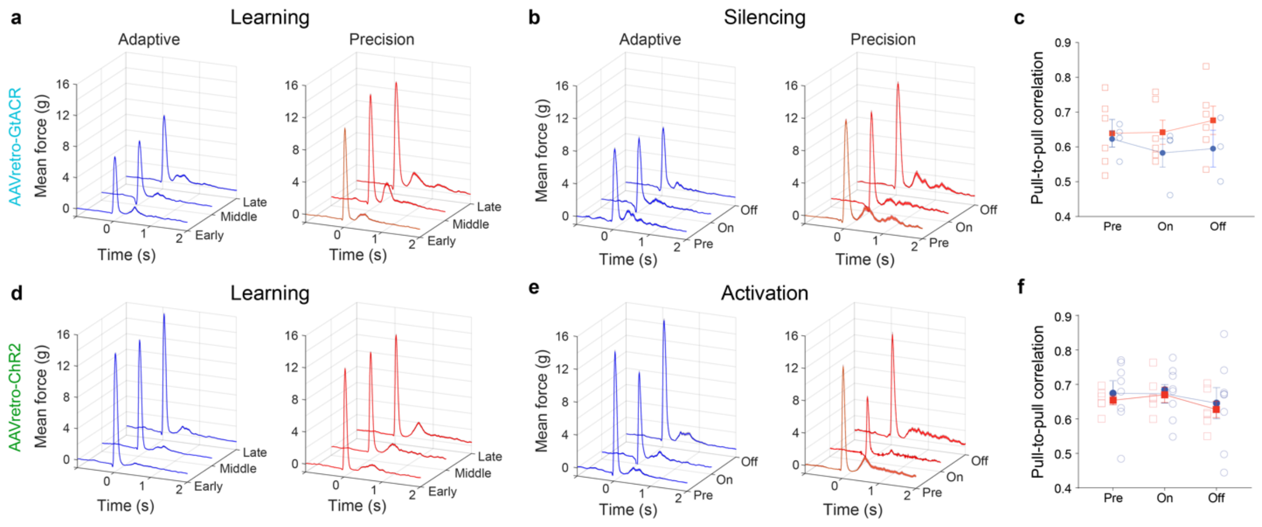

Supplementary Figure 5: Pull force in optogenetic freely moving behavior experiment.

a, d. Mean pull kinetics (mean pull force over learning phases) in adaptive and precision mice during the 2 second reward window (starts at time 0). Neither GtACR2 nor ChR2 expression alters isometric pull force movement over the learning phases in either precision or adaptive groups **b, e.** Isometric pull movement is more robustly disrupted with non-specific activation of ChR2 expressing C7/8 corticospinal neurons than with silencing of those neurons using GtACR2. **c, f.** Average pull-to-pull correlation coefficients were not significantly impaired by optogenetic modulation of C7/8 corticospinal neurons. Data presented as mean ± s.e.m., in panels **a-c** for *n* = 4 mice (adaptive) and 6 (precision); and in panels **d-f** for *n* = 8 (adaptive) and 6 (precision).

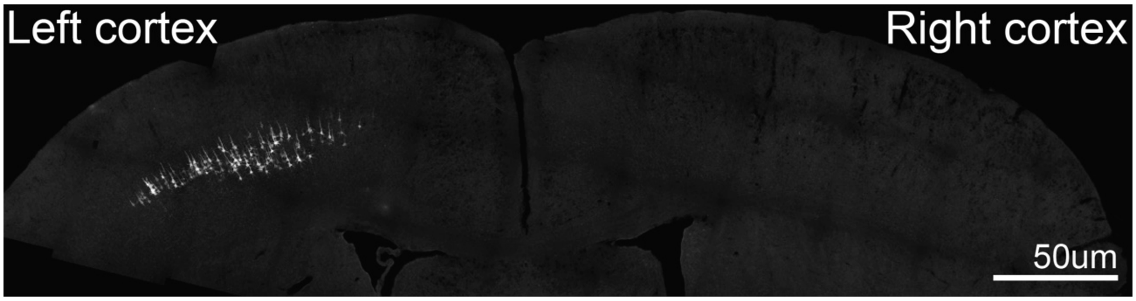

Supplementary Figure 6. Example of *Guillardia theta anion-conducting channelrhodopsin2 (GtACR2)* following unilateral retrograde transduction of right C7/8.

Representative example from 1 experiment, *n* = 4 mice (adaptive) and 6 (precision).

Supplementary Table 1. Number of days for each stage of behavior

		**Freely moving**			**Head-fixed**			**Cannulated (Stimulation)**		**Cannulated (Silencing)**	
	Threshold	Precision	Adaptive	Static	Precision (learners)	Adaptive	Precision (non-learners)	Precision	Adaptive	Precision	Adaptive
Preshaping days (mean ± sem)		3.7±0.2	3.4±0.2	3.8±0.2	3.4±0.7	4.7±0.4	3.6±0.7	4.5±1.4	N/A	5.8 ±0.3	5.3± 0.5
Training days (mean ± sem)	Adaptive	N/A	11.4±0.2	N/A	N/A	16.2±1.5	N/A	N/A	6.5±0.61	N/A	23.0±2.3
	Phase 1	7.4±0.6 (5-20g)	N/A	5.0±0.4 (5g)	5.0±0.5 (5-20g)	N/A	4.6±0.2 (5-20g)	3.1±0.6 (5-20g)	N/A	4.0±1.9	N/A
	Phase 2	0.9±0.2 (8-20g)	N/A	1.5±0.2 (8g)	2.6±0.5 (8-20g)	N/A	2.6±0.4 (8-20g)	1±0 (8-20g)	N/A	3.2±0.8	N/A
	Phase 3	8.6±1.2 (13-19g)	N/A	4.0±0.5 (13g)	8.8±1.7 (13-19g)	N/A	10.6±2.8 (13-19g)	4.8±0.7 (13-19g)	N/A	4.1 ± 2.9	N/A
	Phase 4	34.7±2.0 (15-18g)	N/A	7.0±1.1 (15g)	22.8±4.0 (15-18g)	N/A	22.8±4.4 (15-18g)	18±1.2 (15-18g)	N/A	20.8±0.3	N/A
Postinjury days (mean ± sem)		14.1±0.1	10.0±0.0	12.4±0.9	10.4±0.7	12.2±1.3	9.3±0.8	N/A	N/A	N/A	N/A

Supplementary Table 2. Percentage of total pulls in the reward window

	Freely moving			Head-fixed			Cannulated (Stimulation)		Cannulated (Silencing)	
	Precision (Learners)	Adaptive	Static	Precision (Learners)	Adaptive	Precision Non-(learners)	Precision	Adaptive	Precision	Adaptive
Early	28.4%	60.9%	60.7%	32.9%	63.0%	38.4%	27.0%	61.2%	33.4%	58.5%
Middle	35.8%	61.2%	67.9%	44.9%	63.2%	40.2%	36.9%	63.0%	38.5%	55.5%
Late	48.0%	64.4%	84.1%	51.3%	63.9%	16.7%	47.6%	66.3%	47.4%	57.4%
Postinjury	32.3%	72.1%	68.3%	18.6%	65.1%	20.1%	N/A	N/A	N/A	N/A
Laser Off	N/A	N/A	N/A	N/A	N/A	N/A	47.8%	65.2%	46.8%	54.4%
Laser On	N/A	N/A	N/A	N/A	N/A	N/A	18.1%	60.9%	34.0%	58.2%
Laser Off	N/A	N/A	N/A	N/A	N/A	N/A	43.3%	70.9%	45.3%	55.6%

Supplementary Table 3. Number of corticospinal neurons recorded during imaging sessions

	Early	Middle	Late	Post-injury
Precision (Learners)	267	369	366	345
Adaptive	231	160	215	181
Precision (Non-learners)	492	450	216	192

Supplementary 4. Number of FOVs per timepoint for imaging analysis

	Early	Middle	Late	Post-injury
Precision (Learners)	9	9	10	10
Adaptive	8	8	8	6
Precision (Non-learners)	13	13	12	10

Supplementary Table 5. Statistics reporting by figure

Figure number	Statistical test	n	Descriptive stats (AVG, variance)	P value	Degrees of freedom	Bonferroni post-hoc test (P value)
1d	Repeated measures One-way ANOVA	5, 5, 5	mean±sem	0.0198 (red)<br>0.2416 (blue)<br>0.1715 (magenta)	F (2, 8) = 6.731 (red)<br>F (2, 8) = 1.721 (blue)<br>F (2, 7) = 2.263 (magenta)	Red: Early vs late: 0.0337
1e	Repeated measures One-way ANOVA	5, 5, 5	mean±sem	0.0662(red)<br>0.0776 (blue)<br>0.4296 (magenta)	F (2, 8) = 4.037 (red)<br>F (1, 5) = 4.851 (blue)<br>F (2, 7) = 0.9257 (magenta)	
1f	Repeated measures One-way ANOVA	5, 5, 5	mean±sem	0.0065 (red)<br>0.2040 (blue)<br>0.6400 (magenta)	F (1, 5) = 22.13 (red)<br>F (1, 5) = 2.188 (blue)<br>F (1, 4) = 0.2687 (magenta)	Red: Early vs late: 0.0051
3d	Repeated measures One-way ANOVA	8, 9, 12	mean±sem	<0.0001 (red)<br>0.6302 (blue)<br>0.0019 (gray)	F (1, 10) = 41.18 (red)<br>F (1, 12) = 0.3817 (blue)<br>F (2, 20) = 9.190 (gray)	Red: Early vs late: 0.0003<br>Gray: Early vs late: 0.0047
3e	Repeated measures One-way ANOVA	8, 9, 12	mean±sem	0.0043 (red)<br>0.4516 (blue)<br>0.2690 (gray)	F (1, 10) = 12.09 (red)<br>F (2, 14) = 0.7961 (blue)<br>F (1, 15) = 1.388 (gray)	Red: Early vs late: <0.0001
3f	Repeated measures One-way ANOVA	8, 9, 12	mean±sem	0.3149 (red)<br>0.0064 (blue)<br>0.4966 (gray)	F (2, 11) = 1.242 (red)<br>F (2, 15) = 7.589 (blue)<br>F (2, 17) = 0.6483 (gray)	Blue: Early vs late: 0.0104
3j	Paired two-tailed t test	8, 9, 5	mean±sem	0.0100 (red)<br>0.0025 (blue)<br>0.0826 (gray)	df = 7, t = 3.496 (red)<br>df = 8, t = 4.329 (blue)<br>df = 4, t = 2.304 (magenta)	
3k	Paired two-tailed t test	8, 9, 5	mean±sem	0.2249 (red)<br>0.0014 (blue)<br>0.3546 (gray)	df = 7, t = 1.331 (red)<br>df = 8, t = 4.767 (blue)<br>df = 4, t = 1.046 (magenta)	
3l	Paired two-tailed t test	8, 9, 5	mean±sem	0.0023 (red)<br>0.0002 (blue)<br>0.0121 (gray)	df = 7, t = 4.669 (red)<br>df = 8, t = 6.517 (blue)<br>df = 4, t = 4.356 (magenta)	
4a	Paired two-tailed t test	5, 6, 5	mean±sem	0.0063 (red)<br>0.3719 (blue)<br>0.0727 (magenta)	df = 4, t = 5.257 (red)<br>df = 3, t = 0.3585 (blue)<br>df = 3, t = 2.718 (magenta)	
4b	Paired two-tailed t test	5, 6, 5	mean±sem	0.0057 (red)<br>0.0940 (blue)<br>0.0926 (magenta)	df = 4, t = 5.402 (red)<br>df = 4, t = 2.187 (blue)<br>df = 3, t = 2.439 (magenta)	
4c	Paired two-tailed t test	5, 6, 5	mean±sem	0.0002 (red)<br>0.1278 (blue)<br>0.2277 (magenta)	df = 4, t = 13.59 (red)<br>df = 4, t = 1.916 (blue)<br>df = 3, t = 1.512 (magenta)	
5c	Paired two-tailed t test	6, 4	mean±sem	0.0239 red)<br>0.1416 (blue)	df = 5, t = 3.204<br>df = 3, t = 1.983	
5f	Paired two-tailed t test	6, 8	mean±sem	0.0048 (red)<br>0.4308 (blue)	df = 5, t = 4.814<br>df = 7, t = 0.8358	

All continuous data tested with parametric tests and data assumed to be a Gaussian distribution. Two tailed calculations used in paired experiments. Repeated-measures ANOVA used to test longitudinal behavioral studies, with post-hoc Bonferroni correction on appropriate comparisons.
