# Supplementary material for: Task-specific modulation of corticospinal neuron activity during motor learning in mice
Source: Nat Commun. 2023 May 11;14:2708. doi: 10.1038/s41467-023-38418-4 (PMC10175564; doi:10.1038/s41467-023-38418-4)
Supplement: Supplementary file 3 — Reporting Summary [file 41467_2023_38418_MOESM3_ESM.pdf]

nature portfolio

Corresponding author(s): Edmund Hollis

Last updated by author(s): Apr 18, 2023

Reporting Summary

Nature Portfolio wishes to improve the reproducibility of the work that we publish. This form provides structure for consistency and transparency in reporting. For further information on Nature Portfolio policies, see our Editorial Policies and the Editorial Policy Checklist.

Statistics

For all statistical analyses, confirm that the following items are present in the figure legend, table legend, main text, or Methods section.

n/a	Confirmed	
☐	☒	The exact sample size (*n*) for each experimental group/condition, given as a discrete number and unit of measurement
☐	☒	A statement on whether measurements were taken from distinct samples or whether the same sample was measured repeatedly
☐	☒	The statistical test(s) used AND whether they are one- or two-sided<br>*Only common tests should be described solely by name; describe more complex techniques in the Methods section.*
☐	☒	A description of all covariates tested
☐	☒	A description of any assumptions or corrections, such as tests of normality and adjustment for multiple comparisons
☐	☒	A full description of the statistical parameters including central tendency (e.g. means) or other basic estimates (e.g. regression coefficient) AND variation (e.g. standard deviation) or associated estimates of uncertainty (e.g. confidence intervals)
☐	☒	For null hypothesis testing, the test statistic (e.g. *F*, *t*, *r*) with confidence intervals, effect sizes, degrees of freedom and *P* value noted<br>*Give P values as exact values whenever suitable.*
☒	☐	For Bayesian analysis, information on the choice of priors and Markov chain Monte Carlo settings
☒	☐	For hierarchical and complex designs, identification of the appropriate level for tests and full reporting of outcomes
☐	☒	Estimates of effect sizes (e.g. Cohen's *d*, Pearson's *r*), indicating how they were calculated

Our web collection on statistics for biologists contains articles on many of the points above.

Software and code

Policy information about availability of computer code

Data collection	Data were collected using ThorImage and ThorSync (version 3.2), MotoTrak 2.0 (64-bit)
Data analysis	All software used to analyze data are described in the Method section: ImageJ (version 2.0.0), MATLAB scripts (R2017b), Python (version 3.6.8), Conda (4.10.3), Scikit-learn Python module (version 0.24.2). Illustrations were generated with Adobe illustrator (version 25.2.1). GraphPad Prism (version 9.1.0) was used for statistical analysis.

For manuscripts utilizing custom algorithms or software that are central to the research but not yet described in published literature, software must be made available to editors and reviewers. We strongly encourage code deposition in a community repository (e.g. GitHub). See the Nature Portfolio guidelines for submitting code & software for further information.

Data

Policy information about availability of data

All manuscripts must include a data availability statement. This statement should provide the following information, where applicable:

- Accession codes, unique identifiers, or web links for publicly available datasets
- A description of any restrictions on data availability
- For clinical datasets or third party data, please ensure that the statement adheres to our policy

Datasets generated during this study are available in the public repository https://doi.gin.g-node.org/10.12751/g-node.sbrmy3

Human research participants

Policy information about studies involving human research participants and Sex and Gender in Research.

Reporting on sex and gender	N/A
Population characteristics	N/A
Recruitment	N/A
Ethics oversight	N/A

Note that full information on the approval of the study protocol must also be provided in the manuscript.

Field-specific reporting

Please select the one below that is the best fit for your research. If you are not sure, read the appropriate sections before making your selection.

[x] Life sciences [ ] Behavioural & social sciences [ ] Ecological, evolutionary & environmental sciences

For a reference copy of the document with all sections, see nature.com/documents/nr-reporting-summary-flat.pdf

Life sciences study design

All studies must disclose on these points even when the disclosure is negative.

Sample size	Sample sizes were estimated using power analysis with an alpha of 0.05 and beta of 0.2. Predicted effect sizes were based on prior work in the field using similar animal models (Hollis et al. Nat Neurosci 2016, Li and Hollis J. Neurosci 2021) and ranged from 30-40%.
Data exclusions	Twenty three animals were excluded in total (15 males, 8 females). Ten animals were excluded due to surgical complications, nine animals due to poor learning, three animals due to transduction failure, and one animal did not complete the training.
Replication	Experiments were performed once for each tested condition.
Randomization	The animals were randomly assigned to experimental groups.
Blinding	All statistical analysis was completed by an investigator blind to the data collection. Blinding to distinct training paradigms was not feasible as each paradigm (adaptive, precision, static thresholds) required distinct behavioral shaping regimens.

Reporting for specific materials, systems and methods

We require information from authors about some types of materials, experimental systems and methods used in many studies. Here, indicate whether each material, system or method listed is relevant to your study. If you are not sure if a list item applies to your research, read the appropriate section before selecting a response.

Materials & experimental systems

n/a	Involved in the study	
[ ]	[x]	Antibodies
[x]	[ ]	Eukaryotic cell lines
[x]	[ ]	Palaeontology and archaeology
[ ]	[x]	Animals and other organisms
[x]	[ ]	Clinical data
[x]	[ ]	Dual use research of concern

Methods

n/a	Involved in the study	
[x]	[ ]	ChIP-seq
[x]	[ ]	Flow cytometry
[x]	[ ]	MRI-based neuroimaging

Antibodies

Antibodies used	Rabbit anti-PKCγ (1:100; Santa Cruz; catalog #sc-211; RRID: AB_632234), rabbit anti RFP (1:100; Rockland; catalog #600-401-379; lot #46510; RRID: AB_2209751), Goat anti-rabbit IgG Alexa Fluor 488 (Invitrogen; catalog #A11008, lot #2382186), Donkey anti-rabbit IgG Alexa Fluor 594 (Invitrogen; catalog #A32754, lot #VB292347).
Validation	For rabbit anti-PKCγ: Liu et al., Nature. 2018 (https://doi.org/10.1038/s41586-018-0515-2), Liu et al., Nat Neurosci. 2010 (doi:10.1038/nn.2603), and Hollis et al., Nat Comms. For rabbit anti-RFP: This product was prepared from monospecific antiserum by

immunoaffinity chromatography using Red Fluorescent Protein (Discosoma) coupled to agarose beads followed by solid phase adsorption(s) to remove any unwanted reactivities. Expect reactivity against RFP and its variants: mCherry, tdTomato, mBanana, mOrange, mPlum, mOrange and mStrawberry. Assay by immunoelectrophoresis resulted in a single precipitin arc against anti-Rabbit Serum and purified and partially purified Red Fluorescent Protein (Discosoma). No reaction was observed against Human, Mouse or Rat serum proteins.

Animals and other research organisms

Policy information about studies involving animals; ARRIVE guidelines recommended for reporting animal research, and Sex and Gender in Research

Laboratory animals	Experiments were conducted on adult female and male C57BL6/J Ai14 Rosa26-LSL-tdTomato and C57BL/6J mice (5.83 ± 0.35 weeks of age). Mice were housed on a 12 hr reverse light cycle.
Wild animals	No wild animals were used in this study.
Reporting on sex	84 total mice of both sexes (42 males, 42 females) were randomly assigned to different groups, across all experiments. 61 mice (27 males, 34 females) were used in final reporting after the 23 excluded as described above. Statistical effects across treatment were independent of sex, there were no trends for sex differences across any experiment (either with Kolmogorov-Smirnov or Mann Whitney tests, as appropriate). Therefore, no further animals were added to test for sex differences.
Field-collected samples	No field-collected samples were used in this study.
Ethics oversight	All procedures and surgeries were approved by the Weill Cornell Medicine Institutional Animal Care and Use Committee.

Note that full information on the approval of the study protocol must also be provided in the manuscript.
